# A Radiometric Technique for Monitoring the Desulfurization Process of Blister Copper

**DOI:** 10.3390/s21030842

**Published:** 2021-01-27

**Authors:** Alejandro Vásquez, Francisco Pérez, Maximiliano Roa, Ignacio Sanhueza, Hugo Rojas, Victor Parra, Eduardo Balladares, Roberto Parra, Sergio Torres

**Affiliations:** 1 Metallurgical Engineering Department, University of Concepción, Edmundo Larenas 285, Concepción CCP4070386, Chile; maxiroa@udec.cl (M.R.); hugorojas@udec.cl (H.R.); vparras@udec.cl (V.P.); eballada@udec.cl (E.B.); rparra@udec.cl (R.P.); 2 Electrical Engineering Department, University of Concepción, Edmundo Larenas 219, Concepción CCP4070386, Chile; francisperez@udec.cl (F.P.); ignacio.sanhueza.ruiz@gmail.com (I.S.); sertorre@udec.cl (S.T.)

**Keywords:** spectral measurements, spectroscopy, optical sensors, emissivity, sensing, blister copper, pyrometallurgy, copper converting, copper desulfurization

## Abstract

In this paper, a novel optical technique for following the progress of the blister copper desulfurization process is presented. The technique is based on the changes observed in the continuous spectrum of the visible–near-infrared (VIS–NIR) radiation that the blister melt emits while the chemical reactions of the sulfur elimination process are taking place. Specifically, the proposed technique uses an optical probe composed of an optical fiber, a collimating lens, and a quartz tube, which is immersed in the melt. This optical probe provides a field of view of the blowing zone where the desulfurization reaction occurs. The experimental results show that the melt VIS–NIR total irradiance evolves inversely to the SO_2_ concentration reported by a gas analyzer based on differential optical absorption spectroscopy. Furthermore, the blister copper spectral emissivity as well as the total emissivity observed throughout the process show strong correlation with the sulfur content during desulfurization reaction.

## 1. Introduction

Nowadays, the copper pyrometallurgical industry faces important challenges in terms of economic and environmental sustainability. In order to generate economic benefits of this activity, and due to both the increasing ore complexity and the progressive decrease in the ore grades, it is imperative to augment the production capacity and to improve the production efficiency by developing new technologies for controlling and monitoring the processes [1,2,3,4,5].

In the pyrometallurgical processes, there are sub-stages in which minerals are subject to chemical reactions such as oxidation and desulfurization with the purpose of increasing their purity. The industries of iron, steel, copper, lead, nickel, and zinc are widely known examples in which sulfur and oxygen need to be removed for obtaining the final products [6,7,8,9].

Engaging in experimental research and development in the field of pyrometallurgy is a difficult task due to the intense industrial conditions that compromise the installation of sensing equipment, and the complexity and variety of the physical and chemical phenomena occurring at high temperatures. Specifically, among the factors that must be considered there are multiple chemical reactions, changes in the temperature, and the formation of gases and other intermediate species. In order to overcome these issues, many techniques have been proposed for characterizing and monitoring pyrometallurgical processes based on the measurement of optical properties such as the visible-near-infrared (VIS-NIR) spectral emissions [10,11,12,13,14,15,16,17,18,19,20,21,22,23,24]. On the one hand, in the iron and steel industry, several optical studies based on radiometry and spectral imaging have measured molten-phase temperature, the steel emissivity, and the slag absorption and reflection coefficients. Based on these indicators, it has been possible to develop an optical technique for online slag detection and estimation of the melt depth and the slag layer thickness, which are key for the control and proper monitoring of the siderurgical processes. [17,18,19,20,21]. On the other hand, only few developments based on optical technology for control and monitoring of copper pyrometallurgical processes have been reported [10,11,12,13,14,15,22]. Nowadays, there is only one instrument [22] commercially available for this industry, and it is only intended for monitoring the first stage of the process through the measurement of exhaust gases concentration such as PbS, PbO, and CuOH. As a consequence, many of the methods used to control and monitor the processes in this industry are still qualitative, lack of accuracy, and, in many cases, depend on the decisions taken by an operator [10,11,13,15].

Currently, in the blister copper desulfurization process, the measurement techniques and control instruments that are used are of the batch type in which information is obtained offline by taking physical samples for further analysis. Among the offline analysis techniques, the most used are inductively couple plasma mass spectrometry (ICP-MS), LECO combustion analysis, X-ray diffraction (XRD), X-ray fluorescence (XRF), and microscopy analysis. These techniques provide high-precision compositional information of the samples. However, these techniques have an inherent delay due to the sample preparation for the analysis and the time required for the analysis itself. Therefore, the response times of these high-precision techniques vary from 10 to 30 min, rendering them unsuitable for dynamic processes that require faster response times such as the blister copper desulfurization process. So far, the continuous monitoring of the blister copper desulfurization process is solely performed by a human operator. Based on its own senses, the operator judges the physical characteristics of the melt over the desulfurization process. The precision of its judgment exclusively depends on its experience and is difficult to estimate a priori. Depending on the analyzed variable, e.g., the reactor interior flame color or the surface texture, fracture shape, and color of a melt sample extracted from the furnace, the time employed for the operator to perform the analysis varies from 1 to 10 min. The technique proposed in this study provides continuous monitoring of the process by observing and analyzing the melt radiation over the desulfurization process. The accuracy and suitability of the proposed technique for following the sulfur elimination in the blister copper desulfurization process is supported and verified with a differential optical absorption spectroscopy (DOAS) system and thermocouple temperature measurements in a laboratory experimental setup.

In this paper, a novel technique based on the use of radiometric theory and instrumentation is presented for following the desulfurization process dynamics during the copper pyrometallurgical refining. To the best of our knowledge, this is the first time that a radiometric technique is utilized for characterizing the blister copper desulfurization process, which could lead to the development of new optical sensors for the determination of its progress and for improving the overall control in the pyrometallurgical industry. Specifically, the proposed setup employs a VIS–NIR spectrometer and a fiber optic probe for observing the blowing zone in the bulk. The technique is based on the correlation that exists between the sulfur content, the increase on the melting temperature due to the corresponding highly exothermic desulfurization reaction, and its corresponding VIS–NIR radiation and spectral features. In order to facilitate the analysis of the experimental results, this paper describes in detail a single experiment due to the vast multiplicity of factors that are involved in a desulfurization reaction. The results presented in this paper are in agreement with the behavior observed in a series of experiments in which the blister copper desulfurization process was recreated and monitored using the proposed technique.

### Desulfurization of Blister Copper

The blister copper desulfurization is principally related to two industrial processes of copper pyrometallurgy, which are the copper blowing in the conversion step and the blister copper oxidation in the first stage of pyrorefining. In the copper blowing stage, sulfur is removed as SO_2_ from a melt with mass concentration of 1% sulfur by means of injecting air or oxygen-enriched air. This stage reduces the sulfur mass concentration in the blister to 0.01–0.05%, while keeping an average oxygen mass concentration of 0.1–0.8% [8]. Next, the blister copper is sent to a pyrorefining stage, where the impurities are further reduced until the concentrations of the dissolved sulfur and oxygen reach 0.002–0.005% and 0.05–0.2%, respectively [8,25]. For completeness, a review of the main blister copper desulfurization chemical reactions is presented.

Various authors have reported that the removal of sulfur in the form of SO_2_ by injecting air bubbles into a molten blister copper phase can be explained by two elemental reactions [26,27], one corresponding to an oxygen dissolution reaction in the melt (1) and the other one to a homogeneous desulfurization reaction (2).

(1) $$O_{2_{\left(g\right)}}⟶2{\left[O\right]}_{\mathrm{Cu}}$$

(2) $$2{\left[O\right]}_{\mathrm{Cu}}+{\left[S\right]}_{\mathrm{Cu}}⟶SO_{2_{\left(g\right)}}$$

Based on these two equations, the overall reaction for the desulfurization process can be expressed by the following heterogeneous reaction,

(3) $$\begin{array}{c} {\left[S\right]}_{\mathrm{Cu}}+O_{2_{\left(g\right)}}⟶SO_{2_{\left(g\right)}} \end{array}$$

If oxygen supply continues, and the sulfur contained in the melt has been largely removed, the concentration of dissolved oxygen in the copper increases reaching values between 1.5 to 2.6% [28], depending on the temperature at which the process is carried out, which is typically between 1220 and 1270 °C. The additional supply of oxygen beyond the foregoing point would result in the formation of a new liquid phase of copper oxide immiscible with the metallic copper phase (4), [6,29].

(4) $$\begin{array}{c} 2Cu_{\left(l\right)}+\frac{1}{2}O_{2_{\left(g\right)}}⟶Cu_{2}O_{\left(l\right)} \end{array}$$

Note that in industrial operations, the formation of Cu_2_O_(l)_ is undesirable and very harmful to the furnace refractory walls, and its early detection can prevent severe damages to the furnace and also reduce copper losses in the slag [8].

## 2. Methods and Materials

**Figure 1:**
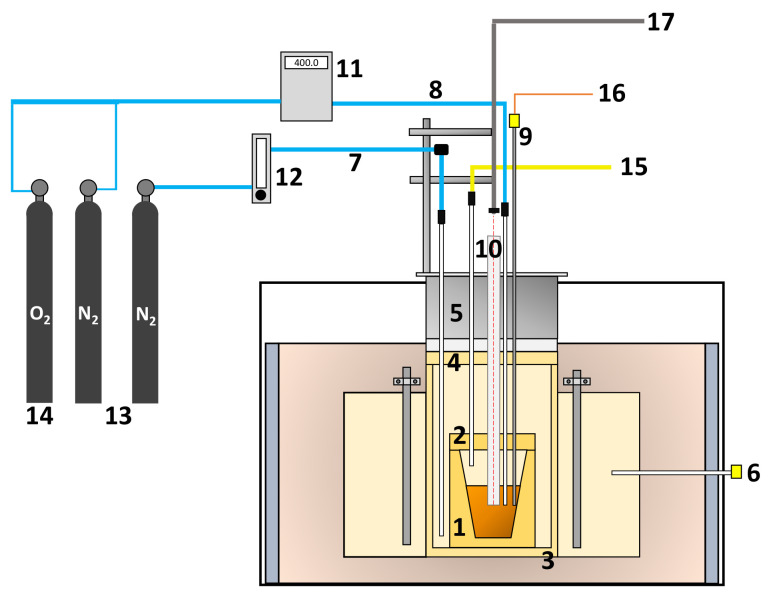
Experimental setup used for the desulfurization experiment: (1) working crucible, (2) working crucible cover, (3) safety crucible, (4) safety crucible cover, (5) furnace cover, (6) S-type thermocouple for controlling the temperature of the furnace, (7) nitrogen supply, (8) oxidant gas supply, (9) K-type thermocouple for monitoring the melt temperature, (10) quartz tube, (11) mass flowmeter, (12) rotameter (13) nitrogen cylinders, (14) air cylinder, (15) gas extraction system, (16) temperature acquisition system, and (17) spectral acquisition system.

**Figure 2:**
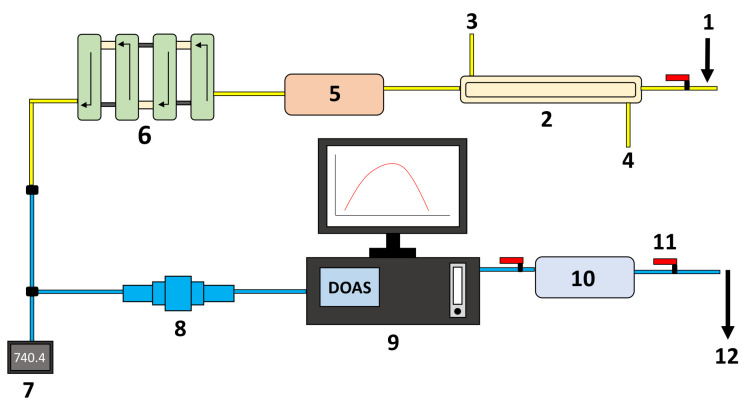
System used to measure the SO_2_ gas produced throughout the experiment. (1) Gas extraction from the furnace, (2) two-pass heat exchanger, (3) compressed air inflow, (4) compressed air outflow, (5) CaSO_4_ dehumidifier, (6) coarse particle settler, (7) pressure transducer, (8) small particle filter, (9) DOAS gas analyzer, (10) silica-gel dehumidifier, (11) stopcock, and (12) gas extraction controlled by Venturi effect.

**Figure 3:**
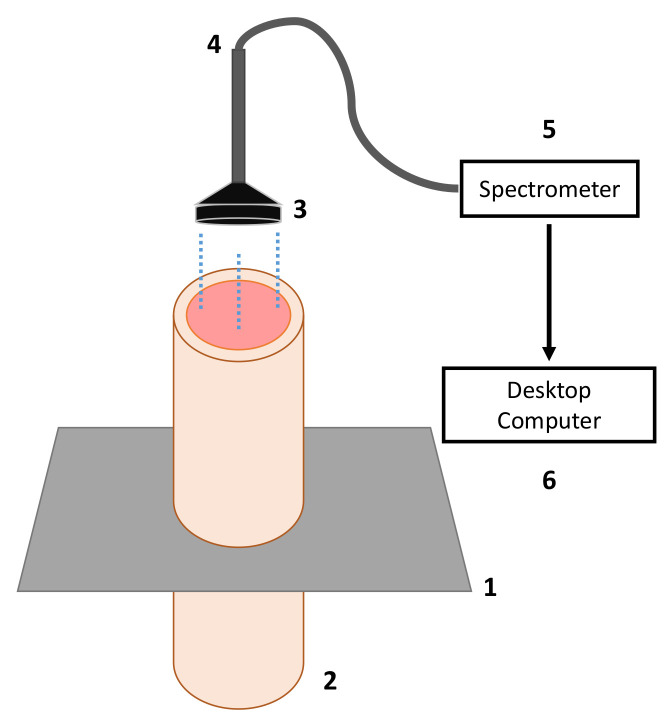
Spectral acquisition system. (1) Furnace cover, (2) quartz tube, (3) collimating lens, (4) fiber optic probe, (5) VIS–NIR spectrometer, and (6) PC for data recording.

The experiment of blister copper desulfurization was carried out using a setup consisting of a high-temperature furnace, a gas injection system, an online DOAS-based SO_2(g)_ analyzer, a temperature acquisition system, and a spectral acquisition system composed of a VIS–NIR spectrometer, a fiber optic probe, a collimating lens and a quartz tube. A general scheme of the components with their interconnection to the reaction furnace, to the DOAS system, and to the spectral acquisition system is presented in Figure 1, Figure 2 and Figure 3, respectively.

### 2.1. Experimental Setup

The high-temperature furnace used in the experiment is a Lindberg electric furnace that has a maximum operation temperature of 1350 °C. The furnace has a cylindrical reaction chamber with 34 cm of height and 43 cm of diameter. In order to measure and control the temperature in the reaction chamber, a lateral S-type thermocouple (Pt–Pt 10% Rh) was connected to a PID controller. Additionally, a cylindrical safety crucible made of alumina was employed in order to protect the physical integrity of the furnace against leaking. This crucible has an internal diameter of 19.5 cm and a height of 34 cm. A working crucible was placed within the safety crucible. The dimensions of the working crucible are 11.5 cm of internal diameter and 14.5 cm of height.

The gas injection and suction system was coupled to the crucible furnace through a stainless steel cover. For the injection of the oxidizing gas, an alumina lance with dimensions of 6 × 4 × 1000 mm was used. The gas injection was controlled by means of a mass flowmeter with an operating range from 0 to 550 mL/min. Prior to the experiment, nitrogen was injected between the safety crucible and the working crucible to displace the oxygen initially contained in the reactive system. This operation was performed throughout the experiment to prevent the entry of oxygen from the outside as the furnace is not a completely closed system. The injection of nitrogen was controlled using a rotameter, and it was introduced to the furnace via a lance of 5 × 2 × 1000 mm. The extraction of the gases generated from the desulfurization process was performed using a lance of 7 × 5 × 1000 mm.

The temperature of the melt over the desulfurization process was measured by means of a K-type thermocouple (Ni 10% Cr-Ni 2% Al) with stainless steel lining. The temperature measurements were recorded using a CompaqDaq temperature acquisition module (manufactured by National Instruments Corporations, Austin, TX, USA).

The composition of the gas produced in the desulfurization stage was registered and monitored using an online DOAS analyzer (manufactured by Unisearch Associates Inc, Concord, ON, Canada). This equipment measures concentration of SO_2_ in volume percentage between 0 and 75%. More specifically, the DOAS gas analyzer uses four simultaneous channels of analysis, each one set for different concentration intervals ranging from low concentration values in the ppm scale to high concentration values in tens of volume percent.

The experimental setup also has a system for cooling, drying, and cleaning the exhaust gases before introducing them to the SO_2_ analyzer, as at the furnace exit they are still hot and humid, and may contain suspended solid particles. As can be observed in Figure 2, the gas cooling was performed by a double-pass heat exchanger coupled to the outside of the crucible furnace for ensuring a gas temperature below 100 °C. A column dehumidifier containing anhydrous calcium sulfate (CaSO_4_) was connected to the exit of the the double-pass heat exchanger. A coarse-particle decanter consisting of four large cross section compartments was added to the outlet of the dehumidifier. Furthermore, a fine-particle filter was installed between the coarse-particle decanter and the inlet of the SO_2_ analyzer.

In order to provide optical access to the melt bulk, a quartz tube was employed as depicted in Figure 1. The tube is 65 mm long and it has an internal diameter of 17 mm and an external diameter of 21 mm. The spectral measurements were acquired using a spectrometer with the help of an optical system consisting of an optical fiber coupled to a collimator. The spectrometer is an HR4000 (manufactured by Ocean Optics Inc., Dunedin, FL, USA), that collects irradiance between 200 nm and 1100 nm in 3648 spectral bands with a spectral resolution of 0.25 nm and quantizes information in 14 bits. The collimating lens is an F810FC-780 (manufactured by Thorlabs Inc., Newton, NJ, USA). The quartz tube was submerged 2.5 cm into the melt and the optical fiber was placed at a distance of 67 cm from the bottom of the quartz tube. In this work, the effective spectral range is trimmed to 650–900 nm due to low signal to noise ratio.

### 2.2. Experimental Design

In order to study the variations of the molten blister copper VIS–NIR spectral emissions in terms of its sulfur content, a desulfurization experiment of a blister-copper sample with sulfur grade of 0.57% was performed. The experiment was carried out in a laboratory electric furnace. Table 1 and Table 2 present a summary of the operating conditions for the furnace and the data acquisition parameters, respectively. The sulfur concentration of the blister copper sample was measured by means of a combustion analysis using a LECO system. In order to prepare the furnace for the desulfurization experiment, the temperature of the reaction chamber was set to 1225 °C. Once the temperature reached the set point, 3.67 kg of blister copper were loaded into a working crucible in the reaction chamber, while nitrogen was used to reduce the oxidation conditions inside the furnace. Once the blister copper was melted and the temperature stabilized at 1225 °C again, the desulfurization process started by the injecting oxidizing gas. The oxidizing gas corresponded to a mixture of 40% of O_2_ and 60% of N_2_, and it was injected into the melt at a rate of 0.42 L/min to promote the desulfurization reaction. This oxidizing atmosphere was maintained throughout the experiment (250 min). The conditions in which the experiment was carried out were chosen so the heat generated by the desulfurization reactions could be easily measured, overcoming the furnace temperature regulation and allowing the sensing equipment to register signals distinguishable from background noise.

The concentration of SO_2_ in the exhaust gases, the melt temperature, and the emission spectra of the blister phase bulk were continuously measured over the whole experiment. The concentration of SO_2_ was monitored using a DOAS-based gas analyzer connected to the furnace gas exit. The percentage of remaining sulfur in the melt is then computed by a mass balance using the melt initial sulfur content, the DOAS readings, and mass flow rate of the exhaust gases. This indirect measurement technique relies on the fact that the furnace is airtight, and thus all the volume of exhaust gases are processed by the DOAS system. The sample temperature was registered using a thermocouple submerged in the melt. The spectral information was collected by means of a digital spectrometer connected to a fiber optic probe with collimating optics to observe inside the melt with the help of a quartz tube partially submerged in the melt, as depicted in Figure 3. The spectral measurements were recorded on a desktop computer every one minute and the spectrometer responsivity was calibrated using a tungsten-halogen light source, Ocean Optics HL-2000-CAL, for the wavelength range of 350 nm to 1.1 μm. Furthermore, the radiometric accuracy of the whole spectral acquisition system (spectrometer, fiber optic, and collimating lens mounted on the furnace as described in Section 2.1 and depicted in Figure 1 and Figure 3) was adjusted by contrasting the emissivity values of liquid copper reported in the literature [30], for temperature values between 1200 and 1250 °C at 650 nm, with the emissivity estimation of a 99.99% Cu sample using the experimental setup described in Section 2.1. A non-reactive atmosphere in the furnace and the same copper mass as in the desulfurization experience were employed for the emissivity estimation of the 99.99% Cu sample.

### 2.3. Spectral Signal Processing

The spectral irradiance emitted by objects at any temperature is described by a continuous spectrum feature that follows a black-body emission, $I_{\mathrm{bb}}\left(\lambda ,T\right)$, as function of wavelength, λ, and object temperature, *T*. This radiation is modeled by Planck’s radiation law, [11]. As real bodies are not ideal emitters, their thermal energy emission efficiency is modeled by means of an emissivity function, ε(λ,T). This emissivity function can be wavelength independent in the case of gray bodies or, more generally, wavelength dependent for real bodies. Therefore, the continuous irradiance spectrum of a real object can be expressed as

(5) $$I_{c}\left(\lambda ,T\right)=\varepsilon \left(\lambda ,T\right)I_{\mathrm{bb}}\left(\lambda ,T\right)$$

(6) $$I_{\mathrm{bb}}\left(\lambda ,T\right)=\frac{2\pi hc^{2}}{\lambda^{5}\left[\exp\left(\frac{hc}{\lambda kT}\right)-1\right]}$$

where *c* is the speed of light, *h* is the Planck’s constant, and *k* is the Boltzmann’s constant. In general, pyrometallurgical processes have a more complex representation that usually includes elemental and molecular emissions in addition to the continuous irradiance spectrum [13]. Currently, such narrow high-energy emissions have neither reported nor observed in the VIS–NIR band for the blister copper desulfurization.

In a dynamic process such as the blister copper desulfurization reaction, the chemical physical and optical properties of the melt vary over time. Thus, throughout the process, both the observed irradiance and emissivity of the melt can be expected to exhibit important variations. In this regard, the use of variables that integrate all their corresponding spectral information into one-dimensional values is very appealing in order to have a better understanding of the overall changes in all the spectral bands due to the process dynamics. Let $\lambda_{min}$ and $\lambda_{max}$ be the minimum and maximum wavelength limits of the spectral acquisition system, then the total observed irradiance is defined as

(7) $$I_{\mathrm{total}}\left(T\right)=\int_{\lambda_{min}}^{\lambda_{max}}I_{c}\left(\lambda ,T\right)d\lambda$$

The total emissivity is correspondingly defined as

(8) $$\varepsilon_{\mathrm{total}}\left(T\right)=\frac{\int_{\lambda_{min}}^{\lambda_{max}}\varepsilon \left(\lambda ,T\right)I_{\mathrm{bb}}\left(\lambda ,T\right)d\lambda }{\int_{\lambda_{min}}^{\lambda_{max}}I_{\mathrm{bb}}\left(\lambda ,T\right)d\lambda }$$

The readings delivered by a digital spectrometer need to be properly calibrated in order to transform the spectra registered in digital counts to radiometric units. Let $I_{\mathrm{raw}}$ be the raw spectrum captured by the spectral acquisition setup described in Section 2.1, then the calibration operation that transforms $I_{\mathrm{raw}}\left(\lambda ,T\right)$ into $I_{c}\left(\lambda ,T\right)$ can be stated as

(9) $$I_{c}\left(\lambda ,T\right)=H\left(\lambda \right)\frac{I_{\mathrm{raw}}\left(\lambda ,T\right)-I_{\mathrm{dark}}\left(\lambda \right)}{T_{i}\;A\;D\left(\lambda \right)}$$

where H(λ) is a gain function in terms of energy units per digital counts and is specific to the sampling optics, $I_{\mathrm{dark}}\left(\lambda \right)$ is the dark current spectrum in digital counts, $T_{i}$ is the integration time, *A* is the collection area, and D(λ) is the wavelength spread of the sensor. The gain function H(λ) is obtained by modeling the responsivity of the spectral acquisition system with the help of a calibration lamp and by calibrating its intensity with the emissivity a sample of 99.99% Cu.

In this work, the melt temperature measured from a thermocouple was used in order to generate the corresponding black-body irradiance for the computation of the spectral emissivity from Equation (5) and the total emissivity from Equation (8).

### 2.4. Error Sources and Limitations

In this experimental system, there are minor error sources corresponding to the accuracy of the employed equipment in addition to the optical system alignment for normal radiation measurements, the optical access cleanliness, the furnace atmosphere control, and the furnace internal temperature control. To overcome these issues, the accuracy of the radiation measurements was ensured by calibrating the whole spectral acquisition system, and the optical access was kept clean by injecting nitrogen and oxygen in the zone where the reaction is measured. Furthermore, before starting the desulfurization process, the furnace atmosphere was kept in a non-oxidizing condition to prevent sulfur loses from the melt, and the furnace internal temperature was regulated by a PID controller with 1 °C of precision. Among the possible error causes, the only one that limits the reliability of the analysis is the fact that the results presented here are only valid for a highly exothermic desulfurization condition that exceeds the thermal regulation of the furnace. If that reaction condition is not met, which could be the case of a slow desulfurization process with little amount of heat generation, the observed changes in the irradiance spectrum resulting from the desulfurization reactions would be competing with the changes induced by the thermal regulation of the furnace. Nonetheless, in the aforementioned scenario, the emissivity analysis of the melt may prove useful to overcome such difficulty.

## 3. Results and Discussion

Here, the experimental results are presented and analyzed in terms of the percentage of sulfur removed in the desulfurization process, the melt temperature and the spectral, and total irradiance dynamics. Additionally, the spectral and total emissivity of the melt are also computed over the desulfurization process. As indicated in Section 2.2, the results presented below were obtained from blister copper desulfurization experiment where the reaction conditions were specially chosen so the heat generated from the chemical reactions could overcome the furnace temperature regulation.

### 3.1. Results

**Figure 4:**
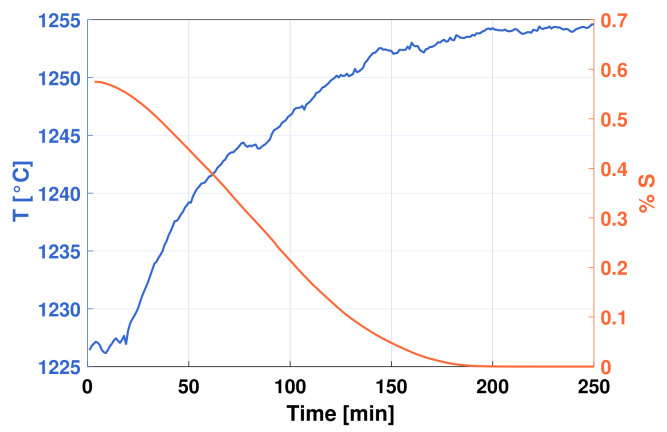
Mass concentration of sulfur and temperature in the blister copper over the desulfurization process.

**Figure 5:**
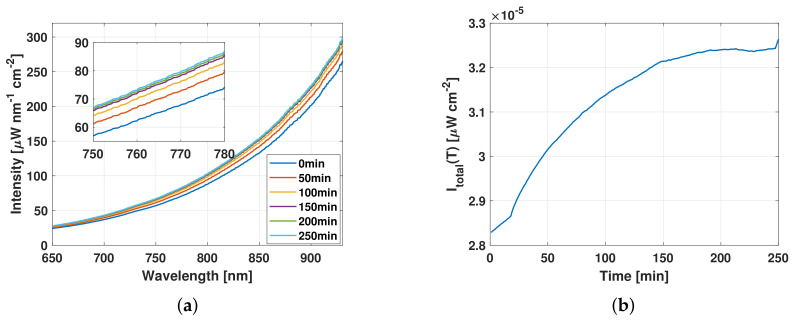
Irradiance of the blister copper over the desulfurization process. (**a**) Calibrated irradiance. (**b**) Total irradiance.

Figure 4 presents the evolution of the blister copper desulfurization process in terms of the sulfur concentration in the melt. As can be observed, the percentage of sulfur in the melt decreases to 0% at 200 min from the beginning of the experiment. The evolution of the melt temperature is shown in Figure 4. The initial temperature of the process was about 1225 °C and by the end of the process it reached 1255 °C in accordance with the exothermic characteristic of the desulfurization reaction that lasted about 250 min. Figure 5a depicts the evolution of melt irradiance spectrum at different times between 0 and 250 min. As can be observed, the irradiance increases in all wavelengths as the desulfurization of blister copper progresses and no spectral interference is detected. Figure 5b displays the total irradiance over the experiment, i.e., the integration of all the measured irradiance at every desulfurization sampling time. An increase of about 15% in the radiation intensity is observed from the beginning to the end of the process. In agreement with the latest findings, the total irradiance, the melt temperature, and the DOAS gas analyzer readings of SO_2_ do not vary significantly in magnitude after the time 200 min, which implies that the desulfurization process has finished.

**Figure 6:**
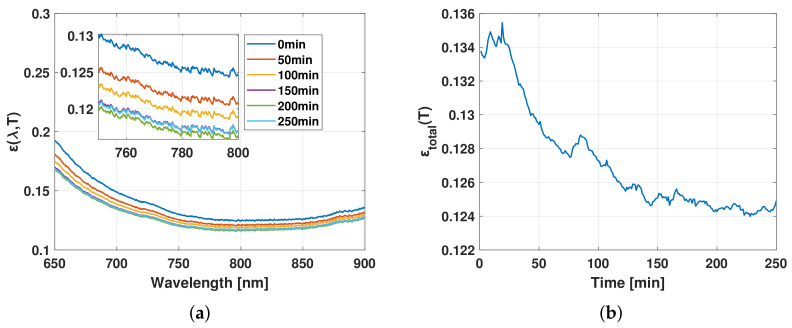
Emissivity of the blister copper over the desulfurization process. (**a**) Spectral emissivity. (**b**) Total emissivity.

Figure 6a shows the evolution of the blister copper emissivity spectrum at different times between 0 and 250 min. As can be observed, the emissivity also decreases in all wavelengths as the desulfurization of blister copper progresses and no spectral interference is detected. Figure 6b displays the total emissivity over the experiment. A decrease in the magnitude of the emissivity curve is observed, finding a difference of 0.01 between the beginning and the end of the process. These values are in accordance with those report in the literature [30,31,32,33,34,35,36,37,38,39]. In agreement with the quantities reported above, the total emissivity does not vary significantly in magnitude after 200 min.

### 3.2. Discussion

The sulfur concentration curve obtained from the measurements of the DOAS system and presented in Figure 4 shows the typical evolution of blister copper desulfurization process [1,26,27]. As can be observed, the sulfur depletion from the melt as SO_2_ occurs at a constant rate when the melt has a high sulfur content (0.57% S), as it occurs in the first 120 min of the experiment (stage 1). However, when the sulfur concentration in the melt becomes scarce, i.e., after 120 min (stage 2), the oxidation (sulfur depletion) slows down. Such a behavior is typical of mixed process in which mass transfer processes are involved [27]. In stage 1, as there is a large amount of sulfur available in the liquid phase, much of the oxygen is reacting very closely at the copper–oxygen interface and the mass transfer processes involved are minimized. In stage 2, caused by the decrease of sulfur in the liquid phase, the thickness of the boundary layer increases and the resistances in the molten phase on the liquid side become more important. The evolution of the melt temperature (Figure 4) is in agreement with the sulfur depletion results, and the exothermic nature of the desulfurization reaction, Equation (3). Specifically, the temperature curve exhibits an inverse behavior compared to the sulfur decreasing curve, which can be used to infer the degree of completion of the desulfurization process. Similar to the sulfur decreasing curve, the major rise in temperature occurs in the first 120 min, and later, the temperature tends to stabilize. The main contribution of this work is that the dynamics of the desulfurization process can be tracked online by measuring the VIS–NIR melt irradiance. The changes in the melt irradiance can be understood with the help of (5), in which the black-body radiation model is directly related to its temperature and the emissivity, which depends on temperature and the melt physical-chemical properties. As the melt spectral intensity uniformly increases at all registered wavelengths with the progress of the desulfurization process, the increase in the melt irradiance is mainly attributed to the exothermal process generated by the desulfurization reactions. The foregoing can be observed in Figure 5a and is even more evident after integrating all the spectral information and analyzing the changes in the irradiance over time, as it is depicted in Figure 5b. The correlation between the temperature and the energy generated from the reaction is evident when comparing the curves in Figure 4 and Figure 5b. Therefore, it is clear that the melt total irradiance can be a useful indicator for determining the moment in which the desulfurization of the blister copper is completed, which can be extremely appealing for industrial applications.

Changes in properties such as spectral emissivity (Figure 6a) and total emissivity (Figure 6b) could be related to the dynamics of the process as it is known that, at a given temperature, a variation in emissivity may be attributed to a change in the blister copper composition. For example, it is well known that many solid metals exhibit difference in their emission properties when they are oxidized [24,40]. Based on this idea, the melt total emissivity using the foregoing calibrated irradiance measurements was computed. For this purpose, the melt temperature (Figure 4) was employed to generate the corresponding black-body irradiance for each measurement. The aforementioned observation can be observed in Figure 6a, as the melt spectral emissivity decreases with the progress of the desulfurization process at all registered wavelengths. This result is even more clear in Figure 6b after integrating all the spectral information and analyzing the changes in the total emissivity over time. The decrease of the total emissivity of the melt as the desulfurization of the melt progresses can be attributed to compositional changes in molten copper as sulfur is eliminated. Therefore, the total emissivity of the melt can also be used as an indicator to predict the progress of the desulfurization process. Moreover, a proper model of the melt emissivity may provide a better understanding of the desulfurization process as a deviation from the model can be attributed to a variation in the melt composition. In this regard, further studies are being performed in order to characterize the variations of the melt emissivity due to changes of process temperature.

The online radiometric technique developed in this work for the monitoring of the blister copper desulfurization process is noninvasive and, by only requiring optical access to the system under study, enables the characterization of the process and account for its changes due to diverse factors such as chemical reactions, temperature variations, and operational parameters. These advantages of the proposed technique make it suitable for many other applications in industrial processes, such as the white metal to blister copper conversion; the flash combustion of copper concentrates; steel refining; monitoring of combustion flames; and, in general, any other processes in which flames, high-temperature phases, or highly exothermic reactions are present.

## 4. Conclusions

In this paper, a novel technique based on the use of VIS–NIR optical sensing has been proposed for following the reaction dynamics during the blister copper desulfurization process. Specifically, it has been demonstrated that the total irradiance of the melt can be used for monitoring the progress of the desulfurization process. Particularly, this is possible because the desulfurization reaction is strongly exothermic and the energy generated from the reaction can be correlated with the melt temperature. Furthermore, the results show that the total emissivity of the blister copper could also be correlated with the sulfur content during the desulfurization reaction. Moreover, a proper model of the melt emissivity may provide a better understanding of the desulfurization process since a deviation from the model can be attributed to a variation in the melt composition.

## 5. Patents

Patents resulting from the work reported in this manuscript: (1) Parra et al., (2020), solicitud CL202003089, INAPI Oficina de Patentes y Marcas de Chile.

## Figures and Tables

**Table 1:** Operating conditions of the furnace.

Variable	Value	Unit
Blister copper mass	3.67	kg
Oxidizing gas flow	0.42	L/min
Nitrogen flow *	10.00	L/min
Suction flow	4.00	L/min
Temperature	1225	°C

* Used to displace oxygen and seal the furnace.

**Table 2:** Data acquisition parameters.

Parameter	Value	Unit
Integration time of the spectrometer	10	ms
Spectrometer sampling time	60	s
Effective spectral range	650–900	nm
Collection area of the fiber probe	7.50 × 10^−1^	cm^2^
Distance of the collimator to the melt	68	cm
Temperature sampling time	60	s
DOAS sampling time	60	s
